# Book Review: Handbook of the Economics of Risk and Uncertainty

**DOI:** 10.3389/fpsyg.2018.00730

**Published:** 2018-05-15

**Authors:** Shabnam Mousavi

**Affiliations:** ^1^Johns Hopkins Carey Business School, Washington, DC, United States; ^2^Max Planck Institute for Human Development, Berlin, Germany

**Keywords:** risk, uncertainty, probability, decision theory, economics

Consider the future as a product of interplay between the states of the nature on one hand and our choices on the other. Perceivably, we can make a particular future come true if we can specify possible outcomes of choices and their relative likelihood. Needless to say, we shall always choose the best option. Economists employ mathematics and logic to make this conviction concrete. Addressing these issues, the *Handbook of the Economics of Risk and Uncertainty* consists of two masterfully crafted prefaces and 14 chapters written by leading economists in theory, empirical, and experimental economics. Below I highlight some central concepts that are examined from different perspectives in many (though not all) chapters. Corresponding chapters and sections in the handbook that discuss each topic are indicated inside parentheses.

Bet on what you believe in. This adage was made concrete by the seventeenth-century representation of beliefs in possible lottery outcomes, artfully complemented three centuries later with the operationalization of the inference of beliefs from observed choices. The latter enabled specifying prior beliefs about future prospects, which was missing from the original Bayesian approach to updating beliefs based on new information (1). Not only could beliefs be represented as specifiable probability distributions, but also the best value or maximum utility could be calculated for rational players whose well-behaved preference rankings were capable of being captured in utility functions. Under risk, where all prospects and their probabilities can be objectively specified, rationality is mainly reflected in the *independence axiom*, which holds that the introduction of a third option, *z*, should not alter an initial preference order between two existing options, *x* and *y*: *x* ≽ *y* → α*x* + (1 − α) *z* ≽ α*y* + (1 − α) *z*. Allais famously produced lottery choices that violate this essential axiom, launching an ongoing line of literature (2).

Moving from risk to situations of uncertainty, probabilities of prospects need to be subjectively assessed. Here the consistency requirement of rationality is preserved by Savage's *sure-thing principle*, which assigns a premium to a given prospect equal to the expected value of the lottery, tantamount to rational risk aversion. However, Ellsberg's famous experiment revealed that not all uncertainties can be captured by subjective probability assignments—giving rise to the concept of *ambiguity* and much follow-up work (2.6, 13, 14.4). Probabilities can be classified according to the distinction not only between objective and subjective but also between aleatory and epistemological. When risk is not objectively known, it can be assessed subjectively, even if it is essentially knowable. On the other hand, economic risk corresponds to the aleatory category of probabilities arising from relative frequencies in repeated trials, whereas uncertainty corresponds to the epistemological category of probabilities, as in degrees of belief. Both meanings seem to lose operational relevance when unknown prospects are involved. This third category of unknowns is referred to as *ignorance* and is material for future research (Preface 2).

Actions do not affect probabilities. This is the main flavor of expected utility calculations. Von Neumann and Morgenstern's (vNM) expected utility theory (EUT) concerns the formation of strategies, mixed and otherwise, for noncooperative, zero-sum situations with no pure equilibrium when uncertainty is objectified as risk (1.2, 3.3). Maximizing a utility function that satisfies the three axioms of vNM—namely, completeness, transitivity, and continuity—is equivalent to choosing the best possible prospect, which by definition is the most preferred option. Savage's contributions to decision theory came in two phases. First, his subjective probability theory provided a framework for constructing relative likelihoods of prospects without preference ordering. Second, his subsequent axiomatic approach to choice under uncertainty defined necessary and sufficient criteria for the joint existence and uniqueness of utility and probability for choices with deterministic consequences in static situations, thereby extending vNM utilities to the subjective level (1.3, 14.1). Further extensions of this idea to dynamic situations by others (2.5, 14.2) dictated that only *naïve* agents who change taste at every stage or *myopic* agents who overlook future stages violate intertemporal consistency, whereas *resolute* agents keep executing the initial plan despite changes in preferences and *sophisticated* agents plan by backward induction based on perfect foresight of their future taste developments, hence acting in a consistent manner along a dynamic path. Thus, resolute and sophisticated agents are rational agents for whom time does not affect planned actions.

The conception of expected utilities can be traced back to the 18th century when, with the introduction of diminishing marginal utility, Daniel Bernoulli remedied the inadequacy of expected value maximization, posed for one by the St. Petersburg paradox. Nonetheless, until the mid-twentieth century, that is, prior to EUT, economists remained focused on analysis of valuation in terms of simple mean-variance (M-V) utility functions, such as *V*(σ, μ) = μ − λ.σ^2^, that rank the agents' preference over random returns (3). This ranking, which is independent of all higher moments, remains to date the main tenet of asset pricing, where the tradeoff between risk and return can be optimized for an investor with given preferences. In model building, these preferences were assumed as given. In the laboratory, risk preferences are elicited in one of three ways (4, 7.2): the proportion of investment in risky versus safe assets in a portfolio, the point at which subjects switch from a risky to a safe gamble on a given menu, and the named selling or buying price for a gamble, which reveals certainty equivalents. The EU ranking coincides with the M-V ranking for normal distribution and generally in the case of a CARA (constant absolute risk aversion) utility function (3.6). Otherwise, when higher moments are significant, such as in skewed distributions, econometrics methods provide nonlinear representations for assessment of risk preferences (4.3).

In sum, the contributors to this handbook view rational decision making as static or dynamic and model it in combination with deterministic, risky, or uncertain consequences. The impetus of the majority of arguments lies in experiments conducted mainly by economists. This collection is deeply rooted in theoretical and axiomatic conceptualizations of decision making under risk and uncertainty with a sprinkling of the psychological studies of heuristics (4.7). This handbook is most useful for cognitive scientists and psychologists who want to learn about the background details of what economists explored and entertained that are now known as central notions of behavioral economics, presented in psychology terminology such as risk aversion, domain of gain versus loss, and reference point. These very concepts, only in different terms, can be traced back to the joint work of Friedman and Savage from 1948 and the subsequent investigations by Harry Markowitz, who observed: “Generally people avoid symmetric bets. This suggests that the curve falls faster to the left of the origin than it rises to the right of the origin.”

## Author contributions

The author confirms being the sole contributor of this work and approved it for publication.

### Conflict of interest statement

The author declares that the research was conducted in the absence of any commercial or financial relationships that could be construed as a potential conflict of interest.

